# Establishment of a novel renal immune prognostic index to predict clinical outcomes in renal cell cancer patients who received surgery

**DOI:** 10.3389/fonc.2025.1608847

**Published:** 2025-12-10

**Authors:** Yu Zhang

**Affiliations:** Department of Nephrology, Second Affiliated Hospital of Harbin Medical University, Harbin, China

**Keywords:** renal cell carcinoma, surgery, immunoglobulin, prognostic model, clinical outcomes

## Abstract

**Objective:**

The aim of this study is to establish a novel Renal Immune Prognostic Index (RIPI) and investigate its predictive ability for the clinical outcomes of renal cell cancer (RCC) patients.

**Methods:**

This multicenter retrospective study included 259 RCC patients who underwent surgical resection at the Second Affiliated Hospital of Harbin Medical University (January 2016–December 2017) as the training cohort, and 350 patients from Harbin Medical University Cancer Hospital during the same period as the external validation cohort. The RIPI was developed using Cox regression with multicollinearity addressed by Lasso regression. The optimal cutoff was determined by Receiver Operating Characteristic (ROC) curve analysis. Survival differences were evaluated with Kaplan–Meier curves, and potential confounding factors were adjusted using Propensity Score Matching (PSM). Model performance and clinical utility were assessed using the concordance index (C-index), calibration curves, time-dependent ROC curves, and decision curve analysis (DCA).

**Results:**

Lasso regression identified prealbumin (PALB), lymphocyte count (LYM), and immunoglobulin M (IgM) as key hematological prognostic parameters. RIPI was constructed as: RIPI = 0.005 × PALB (g/L) + 0.248 × LYM (10^9^/L) + 0.372 × IgM (g/L). The optimal cutoff value of 4.96 stratified patients into low and high RIPI groups. In the training cohort, RIPI showed strong discriminatory ability with an AUC of 0.750, outperforming individual markers and conventional indices. Time-dependent ROC analysis demonstrated consistently higher predictive performance of RIPI across all time points. Kaplan–Meier survival analysis revealed that patients in the low RIPI group had significantly shorter progression-free survival (PFS) and overall survival (OS) (all P < 0.001), and RIPI remained an independent prognostic factor alongside tumor size and TNM stage. After PSM, RIPI continued to demonstrate significant associations with both PFS and OS. In the validation cohort, similar results were observed, with RIPI maintaining robust prognostic value (AUC = 0.723). Nomograms incorporating RIPI achieved good calibration and C-index values, while DCA confirmed its clinical utility.

**Conclusion:**

This multicenter retrospective study demonstrated that RIPI, integrating PALB, LYM, and IgM, provides robust and reproducible prognostic value in RCC patients. RIPI represents a reliable and clinically applicable tool for individualized risk stratification and outcome prediction.

## 1 Introduction

Renal cell carcinoma (RCC) is one of the most common malignancies worldwide (1). In recent years, advances in medical technology and improved screening methods have led to a gradual decline in RCC-related mortality, and RCC has increasingly been regarded as a manageable chronic disease (2). However, its persistently high incidence continues to pose a significant threat to global public health. Currently, surgery remains the cornerstone of RCC treatment, complemented by advancements in targeted therapy, immunotherapy, and radiotherapy. These developments have progressively established a comprehensive treatment strategy centered on surgery, significantly improving patient outcomes (3). Nevertheless, the risk of postoperative recurrence or distant metastasis remains a major challenge for many patients (4). Therefore, early identification of patients at high risk of recurrence and metastasis, along with the development of more precise therapeutic strategies, remains a critical focus in RCC research and clinical practice.

In recent years, significant progress has been made in the research and application of prognostic indicators for tumors. These indicators integrate clinical pathological features, imaging parameters, and molecular biomarkers to provide a scientific basis for assessing patient survival outcomes and formulating individualized treatment strategies (5). Among them, blood-based prognostic indicators have gained increasing attention due to their simplicity, ease of access, cost-effectiveness, and suitability for widespread clinical application (6). These indicators reflect systemic inflammatory states, host immune function, and overall metabolic status, thereby indirectly evaluating disease progression and prognosis (7). For instance, neutrophil-to-lymphocyte ratio (NLR), platelet-to-lymphocyte ratio (PLR), red cell distribution width (RDW), and serum albumin levels can reflect inflammation, coagulation abnormalities, and nutritional or metabolic status, respectively, all of which are closely associated with tumor aggressiveness, growth, and metastatic potential (8). Therefore, these prognostic indicators can reveal the interactions between tumors and the host to a certain extent and provide predictive insights into disease outcomes. However, despite their demonstrated prognostic value across various tumor types, current blood-based prognostic indicators face certain limitations. Most of these indicators are general-purpose and have been applied to multiple malignancies but were not specifically designed for RCC, potentially limiting their ability to fully capture the unique biological characteristics of RCC. Additionally, the rapid development of novel therapeutic strategies, particularly the advent of immune checkpoint inhibitors, has dramatically transformed the landscape of cancer treatment, achieving remarkable clinical efficacy (9). Unlike conventional chemotherapeutic agents that rely on direct cytotoxic effects, ICIs enhance the host’s immune system to combat tumors. This fundamental difference in mechanisms may challenge the predictive capacity of traditional blood-based indicators in the context of modern treatments. Significant progress has also been made in surgical techniques and chemotherapy drugs. Therefore, the development of a novel prognostic indicator tailored specifically for renal cell carcinoma has become a critical focus in current research.

Immunoglobulins, including IgA, IgG, and IgM, are essential components of the immune system, playing a critical role in maintaining immune function (10). They are widely present in blood and bodily fluids, and their levels can directly reflect the overall state of the immune system. Previous studies have demonstrated that immunoglobulin levels hold significant prognostic value in various cancers, with abnormal levels of IgA, IgG, or IgM being closely associated with disease progression, immune imbalance, and clinical outcomes (11). These findings suggest that immunoglobulin levels can serve as important indicators of tumor biology and patient prognosis. A prognostic index based on immunoglobulin levels has the potential to significantly improve the ability to predict patient outcomes and provide a more robust basis for clinical decision-making. Therefore, this study aims to establish a novel Renal Immunoglobulin Prognostic Index (RIPI) for RCC patients, leveraging immunoglobulin levels to systematically evaluate its predictive capacity for clinical outcomes in RCC.

## 2 Methods

### 2.1 Patients

**Figure 1:**
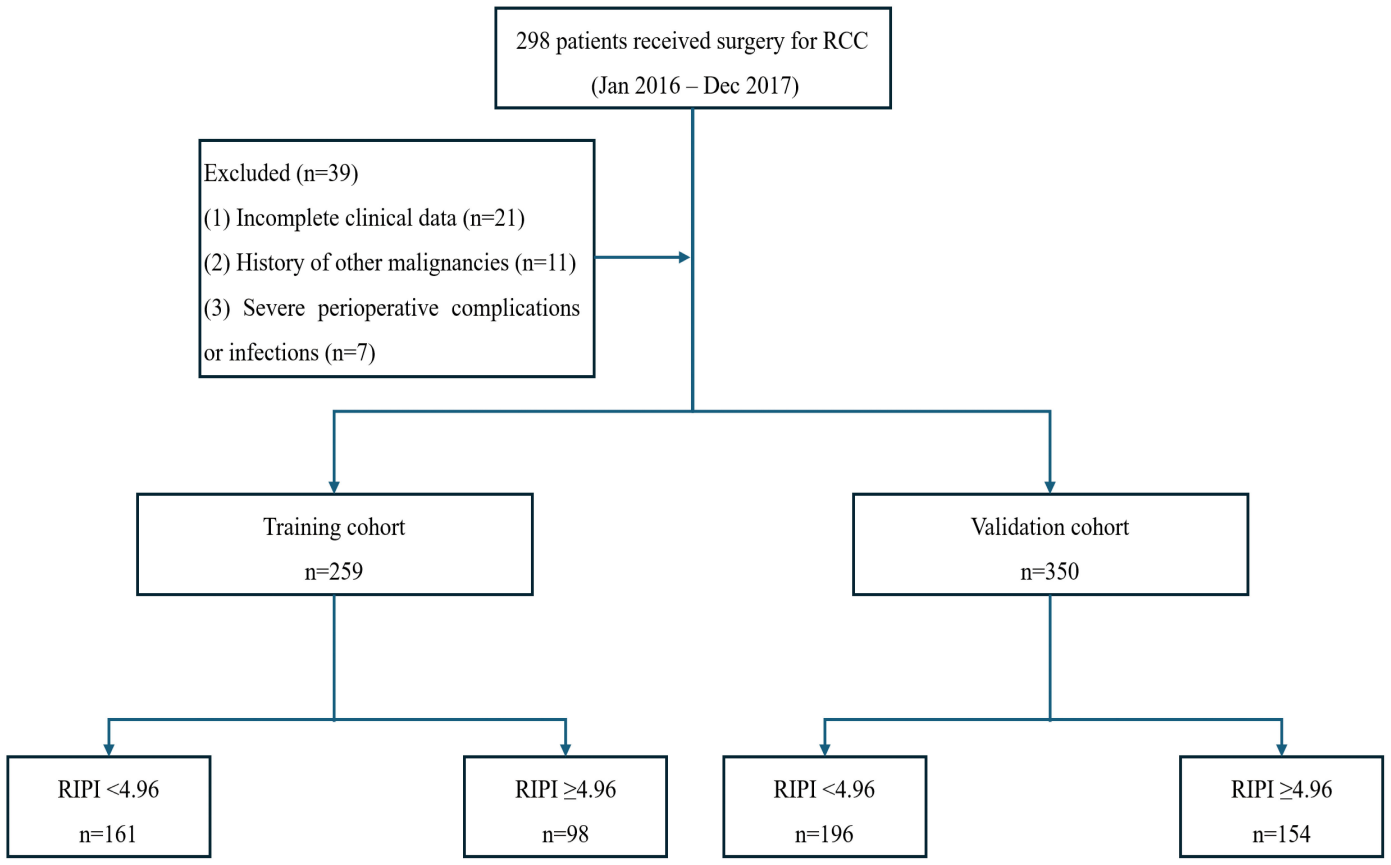
Flow diagram of patient enrollment and group stratification.

This multicenter retrospective study included a total of 259 patients diagnosed with renal cancer who underwent surgical resection at Affiliated Second Hospital of Harbin Medical University between January 2016 and December 2017, defined as the training cohort. During the same period, an additional 350 patients with renal cancer were enrolled from Harbin Medical University Cancer Hospital and were defined as the external validation cohort (**Figure 1**). The inclusion criteria were as follows: (1) histologically confirmed renal cancer; (2) availability of complete clinical and pathological data; (3) no prior systemic therapy, including chemotherapy, radiotherapy, or immunotherapy, before surgery. Patients were excluded if they had (1) incomplete clinical information, (2) a history of other malignancies, or (3) any severe complications or secondary infections during the follow-up period. Written informed consent was signed by all participants, and the study protocol was approved by the Ethics Committee of the Second Affiliated Hospital of Harbin Medical University (No.: YJSKY2023-229). All procedures were conducted in compliance with the applicable guidelines and regulations.

### 2.2 Data collection and follow-up

Patient clinical and pathological data were collected through the electronic medical record (EMR) system, while survival data were obtained via routine telephone follow-ups conducted at regular intervals. Patients were followed from the date of surgery until death or the cutoff date of December 31, 2022. The minimum follow-up duration was 60 months, and the maximum reached approximately 80 months. Follow-up assessments were conducted every 3 months during the first 2 postoperative years and every 6 months thereafter. Follow-up data were collected from outpatient medical records, inpatient hospitalization systems, and regular telephone follow-ups. The median follow-up time was 68 months (range: 60–80 months). A total of 41 patients (15.8%) were censored, including those who were lost to follow-up or alive without progression at the end of the study period. Progression-free survival (PFS) was defined as the time from surgery to the first documented disease progression, based on imaging evidence or clinical diagnosis. Overall survival (OS) was defined as the time from surgery to death from any cause. To establish the RIPI, this study collected comprehensive preoperative routine blood test parameters and immunoglobulin levels from patients. Furthermore, to further evaluate the performance of the novel prognostic index, we calculated NLR, PLR, systemic immune-inflammation index (SII), and prognostic nutritional index (PNI), all of which have demonstrated prognostic value in RCC in previous studies. The calculation formulas are provided in **Table 1**.

**Table 1:** Calculation formulas.

Items	Calculation formulas
NLR	neutrophil (10^9^/L)/lymphocyte (10^9^/L)
PLR	platelet (10^9^/L) /lymphocyte (10^9^/L)
SII	platelet (10^9^/L) × neutrophil (10^9^/L)/lymphocyte (10^9^/L)
PNI	albumin (g/dL) + 5 × lymphocyte (10^9^/L)

NLR, neutrophil-to-lymphocyte ratio; PLR, platelet-to-lymphocyte ratio; SII, systemic immune-inflammation index; PNI, prognostic nutritional index.

### 2.3 Statistical analysis

The normality of continuous variables was assessed using the Shapiro–Wilk test. Non-normally distributed continuous variables were expressed as medians and interquartile ranges (IQRs), and differences between groups were analyzed using the Mann–Whitney U test. Normally distributed variables were summarized as means ± standard deviations (SD), and differences were analyzed using independent-sample *t*-tests. Categorical variables were presented as frequencies and percentages, and group differences were compared using the chi-square test or Fisher’s exact test, as appropriate. Cox proportional hazards regression analysis was performed to construct prognostic indices and identify independent prognostic factors. Least absolute shrinkage and selection operator (LASSO) regression was applied to eliminate potential multicollinearity among variables and select the most informative predictors. Kaplan–Meier (K–M) survival curves and log-rank tests were used to compare survival outcomes between groups. The cutoff value for the Renal Immunoglobulin Prognostic Index (RIPI) was determined using receiver operating characteristic (ROC) curve analysis. The optimal threshold was identified according to the maximum Youden index (J = sensitivity + specificity − 1), which provided the best balance between sensitivity and specificity for predicting survival outcomes. Patients were then classified into low- and high-RIPI groups based on this cutoff value for subsequent survival analysis. Time-dependent ROC (timeROC) analysis was further performed to evaluate and compare the dynamic predictive performance of RIPI and other prognostic indices across different time points. Decision curve analysis (DCA) was conducted to assess the net clinical benefit and potential clinical utility of the RIPI model across a range of threshold probabilities. Propensity score matching (PSM) was applied to reduce selection bias and balance confounding variables between groups. Finally, nomograms were constructed based on independent prognostic factors to estimate 3- and 5-year survival probabilities, and their calibration and discrimination were evaluated using concordance index (C-index) and calibration plots. All statistical analyses were performed using R software (version 4.2.3; Vienna, Austria). A two-sided *P* value < 0.05 was considered statistically significant.

## 3 Results

### 3.1 Patients characteristics

A total of 259 patients were included in the training cohort and 350 in the validation cohort. The mean age was 58.9 years in the training cohort and 59.3 years in the validation cohort. The proportion of male patients was 65.3% and 64.0%, respectively, while the mean BMI was 21.3 and 21.6 kg/m². Based on TNM staging, stage I–III patients comprised the majority in both cohorts. Specifically, in the training cohort, 17.8%, 33.2%, and 46.0% of patients were classified as stage I, II, and III, respectively, while in the validation cohort the corresponding proportions were 20.6%, 29.4%, and 42.6%. Stage IV disease was observed in 3.1% of the training cohort and 7.4% of the validation cohort. No significant differences in clinical or pathological characteristics were observed between the two cohorts (all P > 0.05, **Table 2**).

**Table 2:** Patients characteristics.

Items	Training cohort	Validation cohort	P
	n=259	n=350	
Age (years, Mean [SD])	58.87 (10.13)	59.34 (10.45)	0.620
BMI (Kg/m², Mean [SD])	21.34 (3.38)	21.56 (3.47)	0.480
Gender			0.742
Male	169 (65.3)	224 (64.0)	
Female	90 (34.7)	126 (36.0)	
Tumor size			0.493
<7 cm	161 (62.2)	208 (59.4)	
≥7 cm	98 (37.8)	142 (40.6)	
Tumor necrosis	55 (21.2)	85 (24.3)	0.462
Surgery			0.766
Radical nephrectomy	118 (45.6)	164 (46.9)	
Partial nephrectomy	141 (54.4)	186 (53.1)	
Pathological type			0.683
Clear cell carcinoma	209 (80.7)	278 (79.4)	
Others	50 (19.3)	72 (20.6)	
TNM stage			0.112
I	46 (17.8)	72 (20.6)	
II	86 (33.2)	103 (29.4)	
III	119 (46.0)	149 (42.6)	
IV	8 (3.1)	26 (7.4)	
Fuhrman grade			0.638
G1	78 (30.1)	96 (27.4)	
G2	135 (52.1)	184 (52.6)	
G3	26 (10.0)	40 (11.4)	
G4	20 (7.7)	30 (8.6)	

BMI, body mass index.

### 3.2 Establishment of Renal Immune Prognostic Index in training cohort

All blood parameters included in the model are shown in **Table 3**. OS was the primary outcome, and blood parameters were included as continuous variables in the univariate Cox regression model. The results showed that lactate dehydrogenase (LDH), total protein (TP), albumin (ALB), prealbumin (PALB), white blood cell count (WBC), neutrophil count (NEU), lymphocyte count (LYM), red blood cell count (RBC), hemoglobin (Hb), IgA, IgG, and IgM were all potentially associated with OS (all P < 0.05), as detailed in **Table 4**.

**Table 3:** Blood parameters.

Items	Renal cell carcinoma
	n = 259
ALT (U/L, median [IQR])	18.00(14.00, 24.00)
AST (U/L, median [IQR])	21.00(17.00, 26.00)
γ-GGT (U/L, median [IQR])	17.00(11.00, 28.00)
LDH (U/L, median [IQR])	160.00(143.00, 180.00)
TBIL (μmol/L, median [IQR])	10.73(8.64, 15.23)
DBIL (μmol/L, median [IQR])	3.84(2.86, 5.08)
IDBIL (μmol/L, median [IQR])	7.08(5.42, 10.25)
TP (g/L, mean [SD])	67.31(6.57)
ALB (g/L, mean [SD])	40.94(4.27)
GLOB (g/L, mean [SD])	26.41(3.62)
PALB (mg/L, mean [SD])	273.66(75.11)
Glu (mmol/L, median [IQR])	5.00(4.60, 5.60)
WBC (10^9^/L, median [IQR])	6.38(5.23, 7.59)
NEU (10^9^/L, median [IQR])	3.62(2.80, 4.54)
LYM (10^9^/L, median [IQR])	1.99(1.48, 2.43)
MON (10^9^/L, median [IQR])	0.45(0.37, 0.63)
RBC (10^9^/L, mean [SD])	4.47(0.59)
Hb (10^9^/L, mean [SD])	137.66(23.65)
HCT (10^9^/L, mean [SD])	41.03(6.62)
IgA (g/L, mean [SD])	2.33(0.86)
IgG (g/L, mean [SD])	10.30(2.59)
IgM (g/L, mean [SD])	0.99(0.26)

ALT, alanine aminotransferase; AST, aspartate aminotransferase; γ-GGT, gamma-glutamyl transferase; LDH, lactate dehydrogenase; TBIL, total bilirubin; DBIL, direct bilirubin; IDBIL, indirect bilirubin; TP, total protein; ALB, albumin; GLOB, globulin; PALB, prealbumin; Glu, glucose; WBC, white blood cell count; NEU, neutrophil count; LYM, lymphocyte count; MON, monocyte count; RBC, red blood cell count; Hb, hemoglobin; HCT, hematocrit; IgA, immunoglobulin A; IgG, immunoglobulin G; IgM, immunoglobulin M; BMI, body mass index.

**Table 4:** Cox survival analysis of blood parameters.

Items	Univariate analysis		
	HR	95%CI	*P*
ALT (U/L)	0.989	0.972-1.006	0.212
AST (U/L)	0.997	0.975-1.021	0.819
γ-GGT (U/L)	0.992	0.980-1.005	0.218
LDH (U/L)	1.002	1.001-1.002	0.003
TBIL (μmol/L)	0.964	0.927-1.003	0.073
DBIL (μmol/L)	0.921	0.818-1.038	0.179
IDBIL (μmol/L)	0.952	0.896-1.012	0.113
TP (g/L)	0.975	0.943-0.998	0.043
ALB (g/L)	0.957	0.912-0.989	0.007
GLOB (g/L)	1.011	0.951-1.074	0.724
PALB (mg/L)	0.994	0.984-0.997	<0.001
Glu (mmol/L)	1.062	0.879-1.282	0.534
WBC (10^9^/L)	1.095	1.036-1.177	<0.001
NEU (10^9^/L)	1.119	1.001-1.253	0.046
LYM (10^9^/L)	0.895	0.505-0.995	0.001
MON (10^9^/L)	1.386	0.489-3.831	0.550
RBC (10^9^/L)	0.704	0.498-0.994	0.046
Hb (10^9^/L)	0.990	0.982-0.999	0.021
HCT (10^9^/L)	0.979	0.947-1.011	0.196
IgA (g/L)	0.889	0.647-0.984	0.022
IgG (g/L)	0.543	0.372-0.917	<0.001
IgM (g/L)	0.655	0.411-0.882	<0.001

ALT, alanine aminotransferase; AST, aspartate aminotransferase; γ-GGT, gamma-glutamyl transferase; LDH, lactate dehydrogenase; TBIL, total bilirubin; DBIL, direct bilirubin; IDBIL, indirect bilirubin; TP, total protein; ALB, albumin; GLOB, globulin; PALB, prealbumin; Glu, glucose; WBC, white blood cell count; NEU, neutrophil count; LYM, lymphocyte count; MON, monocyte count; RBC, red blood cell count; Hb, hemoglobin; HCT, hematocrit; IgA, immunoglobulin A; IgG, immunoglobulin G; IgM, immunoglobulin M; HR, hazard ratio; CI, confidence interval.

**Figure 2:**
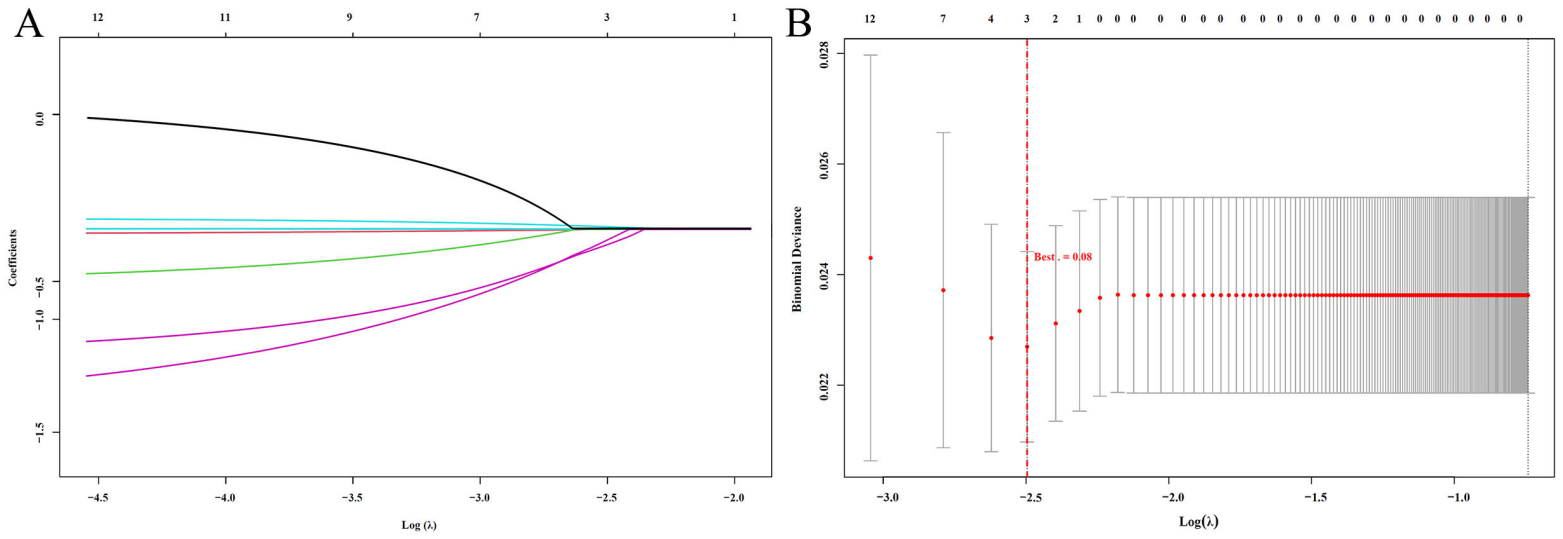
LASSO Regression Analysis **(A)** LASSO coefficient path plot: Shows how regression coefficients change with Log(λ). As λ increases, more coefficients shrink to zero, indicating variable selection; **(B)** LASSO cross-validation error plot: Displays the cross-validation error for different Log(λ) values. The red dashed line marks the optimal λ, balancing model complexity and prediction accuracy.

Subsequently, to eliminate potential multicollinearity, these potential prognostic indicators were included in a Lasso regression model. The optimal lambda value was determined using tenfold cross-validation, with the smallest mean cross-validated error corresponding to a lambda value of 0.08 (**Figures 2A, B**). Ultimately, PALB, LYM, and IgM were identified as significant prognostic factors.

These three indicators were then incorporated into a multivariate Cox regression model, and the Renal Immunoglobulin Prognostic Index (RIPI) was derived based on their beta coefficients. The final RIPI formula is as follows: RIPI = 0.005 × PALB (g/L) + 0.248 × LYM (10^9^/L) + 0.372 × IgM (g/L), shown in **Table 5**. The cutoff value for RIPI, calculated using the ROC curve, was 4.96. Based on this cutoff, patients were grouped into two categories: 161 patients had RIPI <4.96, and 98 patients had RIPI ≥4.96.

**Table 5:** Establishment of RIPI.

Items	β value	HR	95%CI	*P*
PALB	-0.005	0.995	0.992-0.998	<0.001
LYM	-0.248	0.781	0.567-0.932	0.012
IgM	-0.372	0.690	0.435-0.891	<0.001

PALB, prealbumin; LYM, lymphocyte count; IgM, immunoglobulin.

### 3.3 Cox’s survival analysis in training cohort

Cox’s analysis showed that age (P<0.001), BMI (P = 0.038), tumor size (P<0.001), incisal edge (P = 0.023), TNM stage (P<0.001), Fuhrman grade (P = 0.012), and RIPI (P<0.001) were associated with patients’ PFS. Further analysis revealed that age (HR = 1.039, P = 0.011), tumor size (HR = 2.597, P<0.001), TNM stage (HR = 3.001, P<0.001), and RIPI (HR = 0.390, P<0.001) were independent prognostic factors for PFS (**Table 6**).

**Table 6:** Cox’s survival analysis for PFS.

Items	PFS			
	Univariate analysis	*P*	Multivariate analysis	*P*
	HR (95% CI)		HR (95% CI)	
Age	1.055(1.023-1.072)	<0.001	1.039(1.017-1.067)	0.011
BMI	0.925(0.868-0.997)	0.038	0.976(0.835-1.072)	0.229
Gender	0.779(0.539-1.221)	0.384		
Tumor size	3.018(2.074-4.685)	<0.001	2.597(1.758-3.991)	<0.001
Incisal edge	1.734(1.299-2.556)	0.023	1.214(0.929-1.732)	0.448
Tumor necrosis	1.118(0.847-1.690)	0.480		
Radical nephrectomy	1.234(0.727-2,198)	0.330		
Pathological type	0.804(0.618-0.945)	0.103		
TNM stage	3.775(2.763-4.898)	<0.001	3.001(2.427-4.172)	<0.001
Fuhrman grade	1.604(1.171-1.973)	0.012	1.195(0.792-1.380)	0.505
RIPI	0.163(0.084-0.316)	<0.001	0.390(0.122-0.507)	<0.001

In the analysis of OS, age (P = 0.001), BMI (P = 0.042), tumor size (P<0.001), incisal edge (P = 0.019), TNM stage (P<0.001), Fuhrman grade (P = 0.008), and RIPI (P<0.001) were found to significantly influence OS. Further analysis identified tumor size (HR = 2.647, P<0.001), TNM stage (HR = 3.285, P<0.001), and RIPI (HR = 0.318, P<0.001) as independent prognostic factors for OS (**Table 7**).

**Table 7:** Cox’s survival analysis for OS.

Items	OS			
	Univariate analysis	*P*	Multivariate analysis	*P*
	HR (95% CI)		HR (95% CI)	
Age	1.044(1.017-1.068)	0.001	1.006(0.971-1.033)	0.454
BMI	0.938(0.879-0.976)	0.042	0.988(0.892-1.064)	0.270
Gender	0.804(0.676-1.375)	0.384		
Tumor size	3.224(2.362-4.387)	<0.001	2.647(1.893-4.256)	<0.001
Incisal edge	1.796(1.350-2.868)	0.019	1.199(0.838-1.739)	0.528
Tumor necrosis	1.191(0.857-1.707)	0.173		
Radical nephrectomy	1.128(0.841-1,888)	0.630		
Pathological type	0.767(0.442-0.998)	0.077		
TNM stage	3.955(2.918-4.727)	<0.001	3.285(2.683-4.583)	<0.001
Fuhrman grade	1.777(1.284-2.312)	0.008	1.206(0.849-1.537)	0.421
RIPI	0.154(0.073-0.300)	<0.001	0.318(0.125-0.482)	<0.001

### 3.4 Comparison of RIPI-based and clinical prognostic models

**Figure 3:**
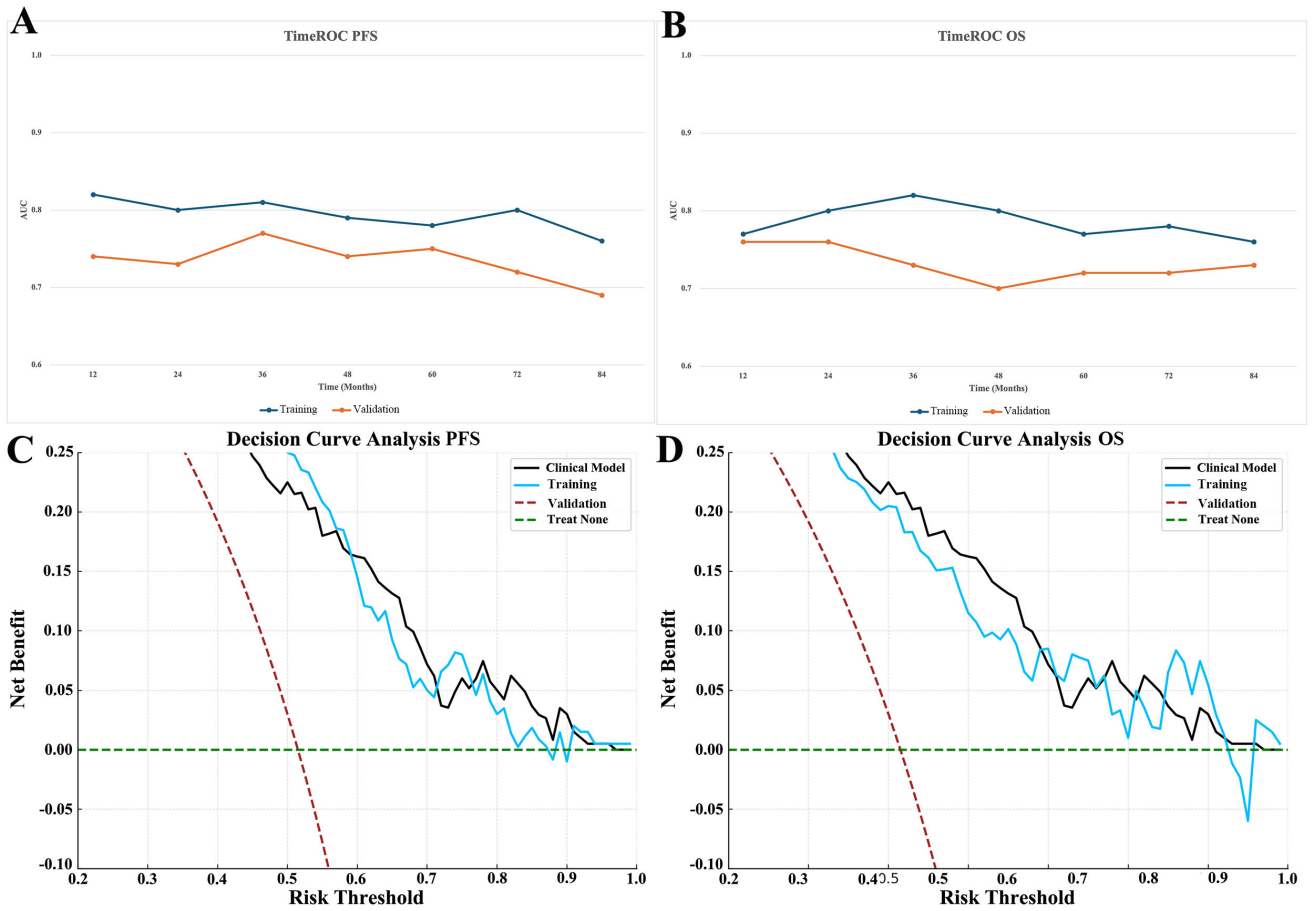
Comparison of prognostic performance between the RIPI model and the clinical model B **(A)** Time-dependent ROC curves for PFS; **(B)** Time-dependent ROC curves for OS; **(C)** Decision curve analysis (DCA) for OS; **(D)** Decision curve analysis (DCA) for PFS .

To further evaluate the prognostic value of RIPI, two multivariate Cox regression models were constructed based on independent prognostic factors: a clinical model (including tumor size and TNM stage) and a RIPI model (including RIPI, tumor size, and TNM stage). The predictive performance of the two models was assessed using timeROC curves, C-index, and decision curve analysis (DCA). As shown in **Table 8**, RIPI, tumor size, and TNM stage were all identified as significant independent prognostic factors in both models (all P < 0.001). TimeROC analysis demonstrated that the RIPI model consistently outperformed the clinical model across multiple time points, showing superior discriminative ability (**Figures 3A, B**). The area under the curve (AUC) values of the RIPI model remained above 0.78 for PFS prediction and were stably above 0.75 for OS prediction. Consistently, the C-index of the RIPI model was higher than that of the clinical model for both PFS (0.78 vs. 0.71) and OS (0.76 vs. 0.69), further supporting the enhanced prognostic discrimination of the RIPI-based approach. Furthermore, DCA curves confirmed that the RIPI model provided greater net clinical benefit than the clinical model within the threshold probability range of 0.3–0.6, suggesting its superior practical utility in guiding clinical decision-making (**Figures 3C, D**).

**Table 8:** Comparison of multivariate Cox regression results between the clinical model and RIPI model for PFS and OS.

Items	PFS	*P*	OS	*P*
	HR (95% CI)		HR (95% CI)	
Clinical Model				
Tumor size	2.597 (1.758–3.991)	<0.001	2.647 (1.893–4.256)	<0.001
TNM stage	3.001 (2.427–4.172)	<0.001	3.285 (2.683–4.583)	<0.001
RIPI Model				
Tumor size	2.109 (1.393–3.202)	<0.001	2.188 (1.452–3.298)	<0.001
TNM stage	2.612 (1.888–3.809)	<0.001	2.901 (2.011–4.078)	<0.001
RIPI	0.390 (0.122–0.507)	<0.001	0.318 (0.125–0.482)	<0.001

### 3.4 Prognostic value of RIPI in training cohort

**Figure 4:**
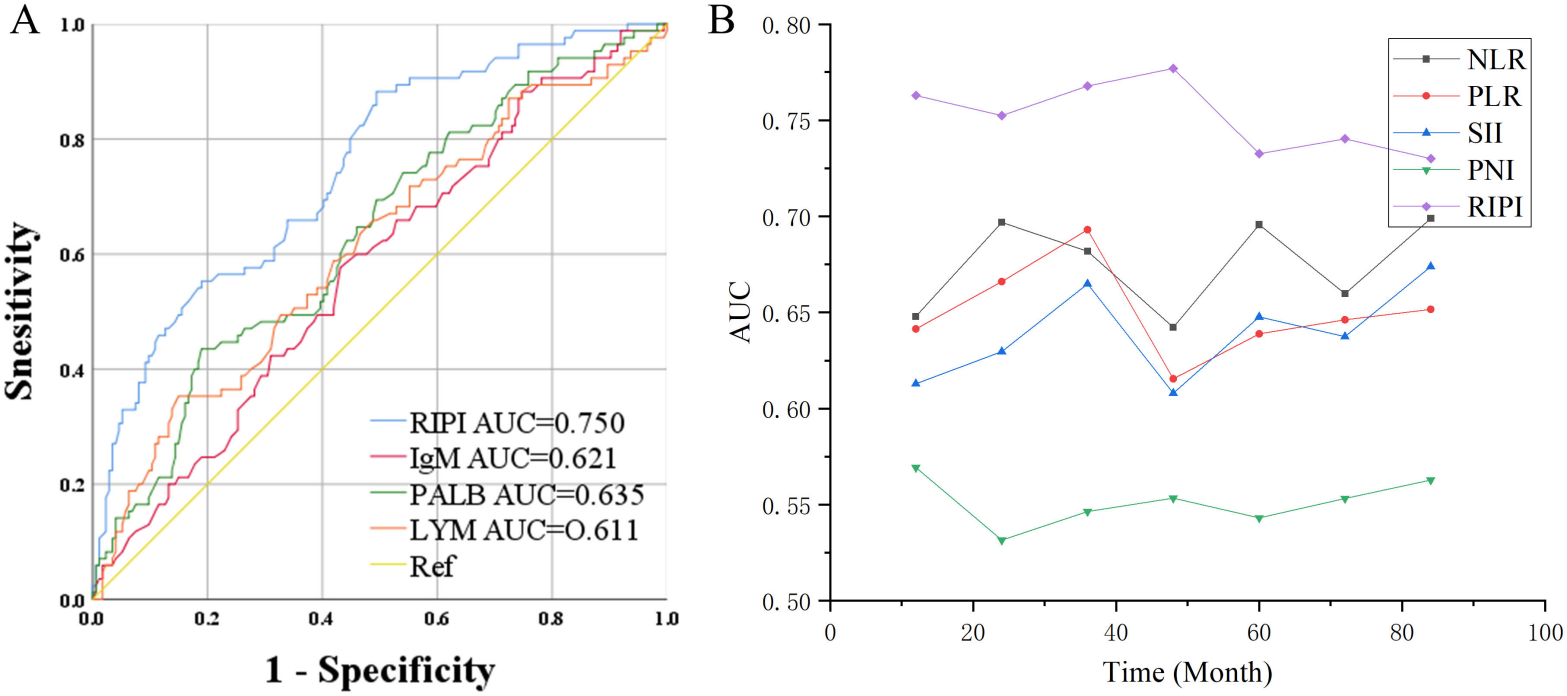
Prognostic value of RIPI. **(A)** ROC curve of RIPI: RIPI has a higher AUC than its component indicators; **(B)** Time-ROC of RIPI: RIPI has the highest AUC at all time points.

ROC curve analysis showed that the AUC values for RIPI, IgM, PALB, and LYM were 0.750, 0.621, 0.635, and 0.611, respectively, with RIPI exhibiting the highest AUC (**Figure 4A**). To compare the performance of RIPI with other prognostic indices, we further generated time-dependent ROC curves. The results demonstrated that RIPI consistently displayed a higher AUC across all time points (**Figure 4B**). To further compare RIPI with other established indices, we constructed a multivariate Cox model including NLR, PLR, and PNI, while excluding SII due to potential collinearity with NLR and PLR. In this analysis, RIPI retained strong prognostic value (**Table 9**).

**Table 9:** Multivariate Cox analysis of RIPI and other indices.

Items	HR (95% CI)	P
RIPI	0.405 (0.268–0.545)	<0.001
NLR	1.068 (1.028–1.110)	0.002
PLR	1.041 (0.995–1.092)	0.078
PNI	0.905 (0.755–1.085)	0.241

RIPI, Renal Immunoglobulin Prognostic Index; NLR, neutrophil-to-lymphocyte ratio; PLR, platelet-to-lymphocyte ratio; PNI, prognostic nutritional index; HR, hazard ratio; CI, confidence interval.

**Figure 5:**
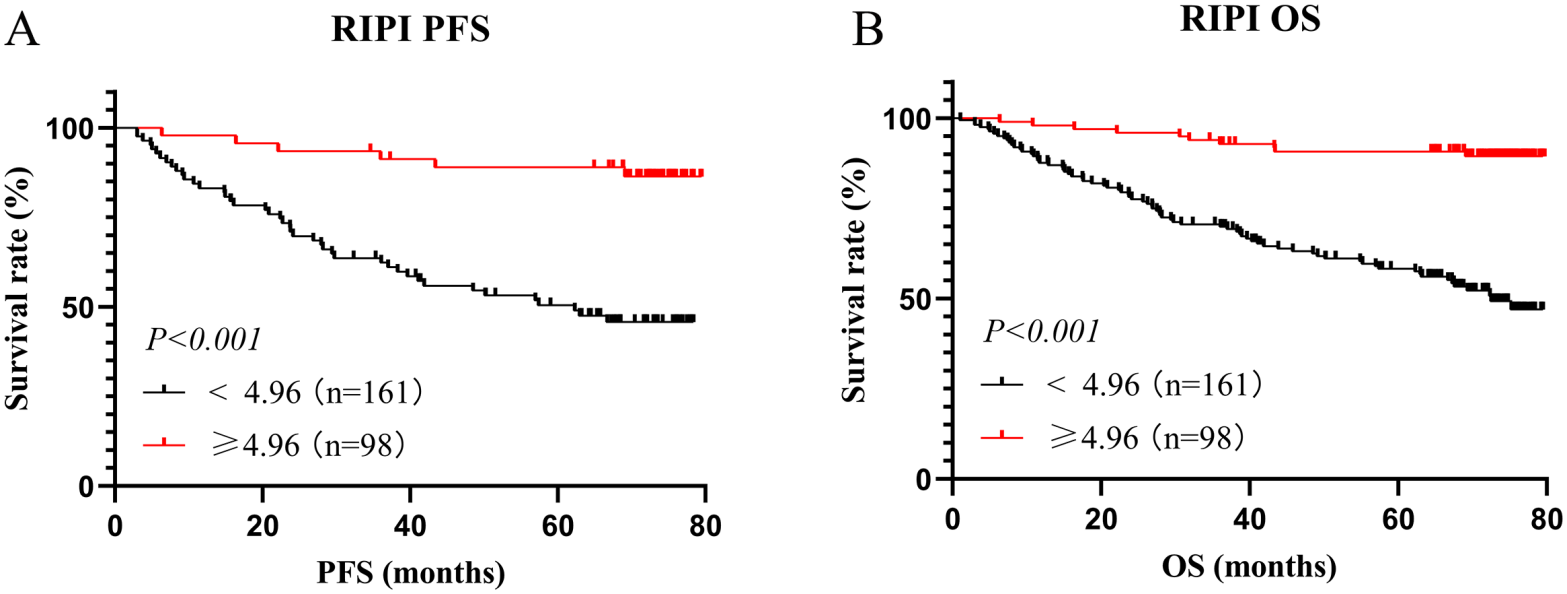
Survival analysis for RIPI. **(A)** Survival analysis for RIPI of PFS: Patients with RIPI <4.96 have a shorter PFS; **(B)** Survival analysis for RIPI of OS: Patients with RIPI <4.96 have a shorter OS.

In this study, there were 161 patients with RIPI <4.96, with a median PFS of 62.27 months and a median OS of 72.53 months. Meanwhile, 98 patients had RIPI ≥4.96, and neither the median PFS nor OS was reached. The results showed that patients with RIPI <4.96 had shorter PFS and OS (χ²=19.29, P<0.001 and χ²=21.41, P<0.001, **Figures 5A, B**).

### 3.5 PSM analysis of RIPI in training cohort

To further eliminate the influence of confounding factors, we performed a PSM analysis of RIPI based on patients’ baseline characteristics and the results from Cox regression analysis. Before PSM, there were 161 patients in the low RIPI group and 98 patients in the high RIPI group. Significant differences were observed between the two groups in terms of age (P = 0.043), BMI (P = 0.032), tumor size (P<0.001), and TNM stage (P<0.001). After matching, each group was reduced to 65 patients, and no significant differences were found between the two groups in terms of any baseline factors (all P>0.05). These results indicate that after PSM, the baseline characteristics between the low and high RIPI groups were better balanced, thereby minimizing potential confounding effects and making the two groups more comparable for further analysis (**Table 10**).

**Table 10:** PSM analysis of RIPI.

Items	Before PSM		*P*	After PSM		*P*
	Low RIPI	High RIPI		Low RIPI	High RIPI	
	n=161	n=98		n=65	n=65	
Age (years), mean (SD)	59.36(10.34)	57.27(10.86)	0.043	58.24(10.19)	58.08(10.22)	0.832
BMI (Kg/m^2^), mean (SD)	20.52(3.69)	22.90(3.71)	0.032	21.92(3.76)	21.37(3.05)	0.721
Gender			0.527			0.893
Male	104(64.6)	65(66.3)		42(64.6)	41(63.1)	
Female	47(35.4)	33(33.7)		23(35.4)	24(36.9)	
Tumor size, n (%)			<0.001			0.127
<7 mm	94(58.4)	67(68.4)		39(60.0)	41(63.1)	
≥7 mm	67(41.6)	32(31.6)		26(40.0)	24(36.9)	
TNM stage, n (%)			<0.001			0.095
I	24(14.9)	22(22.4)		10(15.4)	12(18.5)	
II	49(30.4)	34(34.7)		21(32.3)	21(32.3)	
III	80(49.7)	42(42.9)		34(52.3)	32(49.2)	
IV	8(5.0)	0(0.0)		0(0.0)	0(0.0)	

### 3.6 Survival analysis for RIPI after PSM

**Figure 6:**
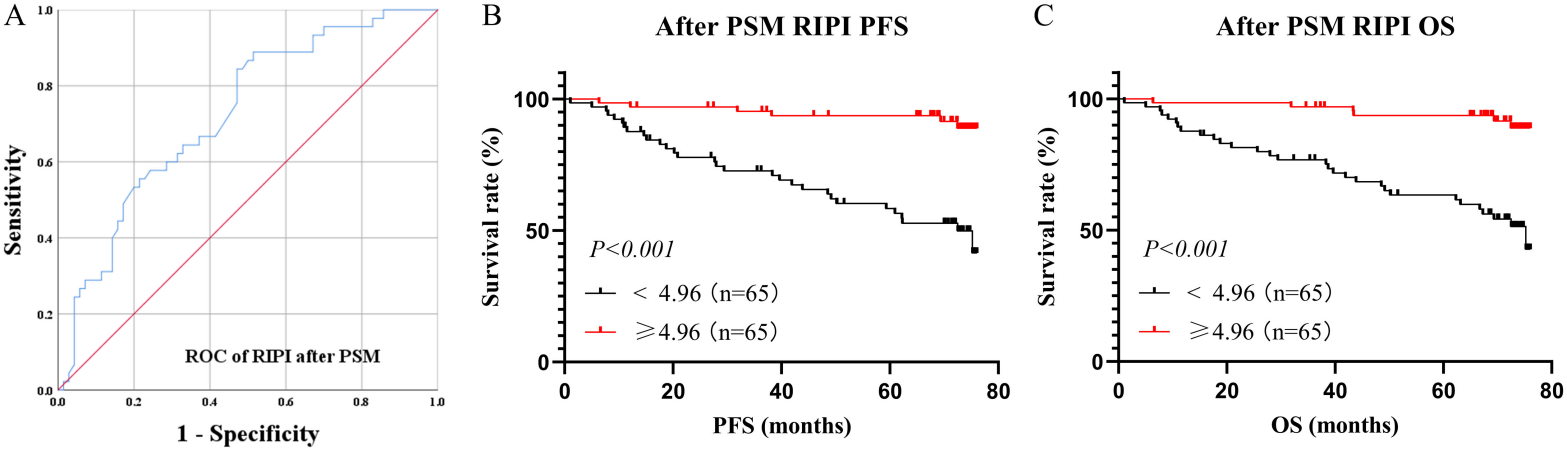
Survival analysis for RIPI after PSM. **(A)** ROC curve of RIPI after PMS: The AUC of RIPI remains at a high level; **(B)** Survival analysis for RIPI of PFS: Patients with RIPI <4.96 have a shorter PFS; **(C)** Survival analysis for RIPI of OS: Patients with RIPI <4.96 have a shorter OS.

After PSM, the ROC curve of RIPI showed an AUC of 0.725, still maintaining a relatively high level (**Figure 6A**). Survival analysis indicated that patients with RIPI < 4.96 had shorter PFS and OS (χ² = 21.25, P < 0.001 and χ² = 23.33, P < 0.001, **Figures 6B, C**).

### 3.7 Nomogram for PFS and OS in training cohort

**Figure 7:**
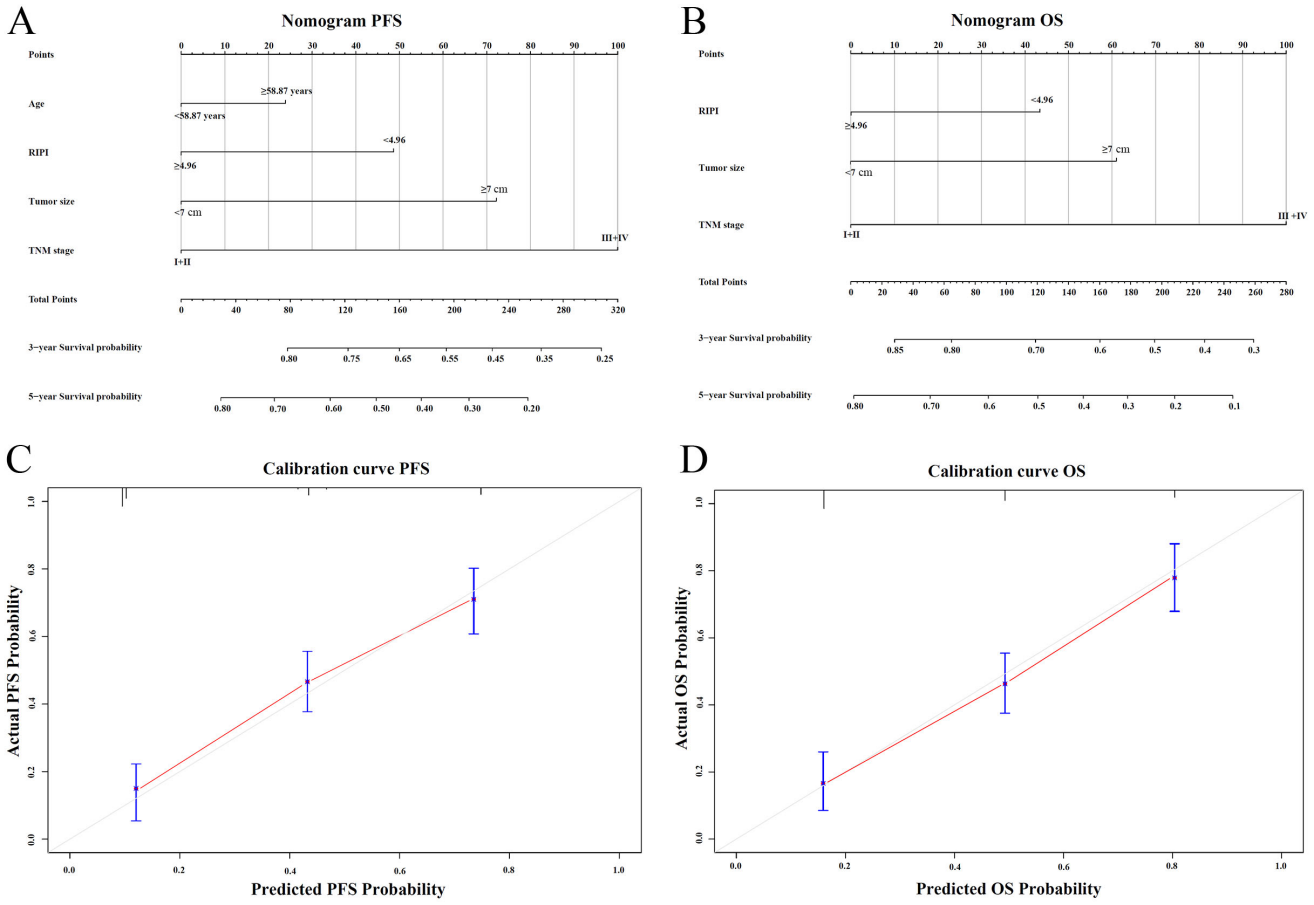
Nomogram and calibration plots for RIPI. **(A)** Nomogram for predicting 3- and 5-year PFS based on independent prognostic factors; **(B)** Nomogram for predicting 3- and 5-year OS based on independent prognostic factors; **(C)** Calibration plot of the nomogram for PFS at 3 and 5 years; **(D)** Calibration plot of the nomogram for OS at 3 and 5 years.

Nomograms for predicting PFS and OS were established based on independent prognostic factors identified in the multivariate Cox regression analysis (**Figures 7A, B**). The C-index of the PFS nomogram was 0.76 (95% CI: 0.71–0.82), and the C-index of the OS nomogram was 0.78 (95% CI: 0.72–0.83), showing good discrimination. Calibration plots showed that the predicted 3- and 5-year survival probabilities were in good agreement with the observed outcomes (**Figures 7C, D**).

### 3.8 Prognostic value of RIPI in the validation cohort

**Figure 8:**
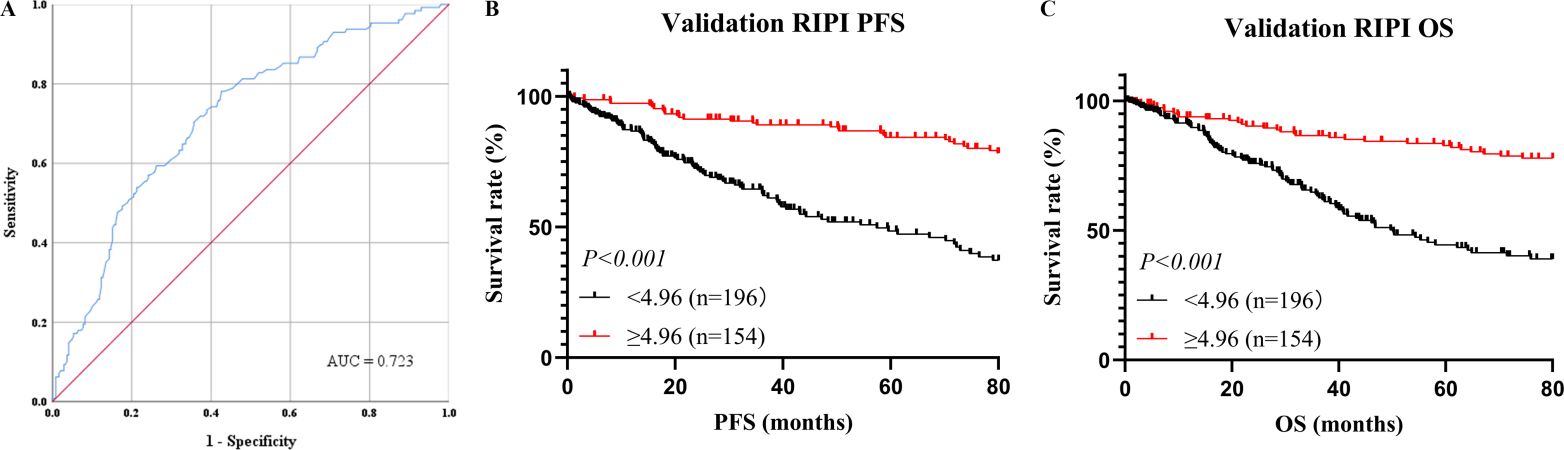
Prognostic value of RIPI in the validation cohort **(A)** ROC curve for overall survival (OS) prediction by RIPI, showing good discriminatory ability (AUC = 0.723); **(B)** Kaplan–Meier curves for progression-free survival (PFS) stratified by RIPI, showing that patients with RIPI <4.96 had significantly shorter PFS; **(C)** Kaplan–Meier curves for overall survival (OS) stratified by RIPI, showing that patients with RIPI <4.96 had significantly shorter OS.

To further validate the prognostic value of RIPI, survival analyses were performed. In the validation cohort, the same cutoff value of 4.96 was applied for risk stratification. Based on this threshold, 196 patients (56.0%) were classified into the low RIPI group, while 154 patients (44.0%) were assigned to the high RIPI group. The ROC curve showed that RIPI had a favorable discriminatory ability for overall survival, with an AUC of 0.723 (**Figure 8A**). Kaplan–Meier analysis demonstrated that patients in the low RIPI group had significantly shorter PFS and OS compared with those in the high RIPI group (PFS: χ²=19.6, P<0.001; OS: χ²=21.3, P<0.001) (**Figures 8B, C**). These findings confirm the robustness of RIPI as a prognostic indicator in patients with RCC.

## 4 Discussion

The development of effective prognostic indicators is of great significance for patients with malignant tumors, especially in the context of the evolving concept of precision medicine. In this study, the Renal Immunoglobulin Prognostic Index (RIPI) was established based on RCC patients, incorporating nutritional, inflammatory, and immune markers, demonstrating high prognostic value. Compared with other indices, RIPI offers significant advantages in predicting the prognosis of RCC patients and effectively identifying those at high risk of recurrence.

In the preliminary analysis, this study identified PALB, LYM, and IgM as significant factors influencing OS, forming the basis for the development of RIPI. PALB is a sensitive marker of nutritional status, reflecting the body’s protein synthesis capacity and nutritional reserves. Compared to traditional nutritional markers such as albumin, PALB has a shorter half-life (approximately 2 days), making it more responsive to changes in the body’s nutritional and metabolic status (12). Moreover, PALB is less affected by external factors such as liver function or inflammation, allowing for a more accurate assessment of recent nutritional levels (13). Low PALB levels are closely associated with malnutrition and poor clinical outcomes in cancer patients (14). In RCC patients, decreased PALB levels may indicate a state of systemic depletion, which not only impairs the immune response but also weakens the body’s ability to combat tumors, thereby exacerbating disease progression. Numerous previous studies have demonstrated the strong prognostic value of PALB. As early as 1978, research teams began exploring the significance of prealbumin, retinol-binding protein, transferrin, and albumin levels in colorectal cancer patients. They found that PALB was the most sensitive prognostic marker among these indicators and demonstrated predictive capability for mortality, typically showing a rapid decline 2–3 months prior to a patient’s death (15). Another study reached a similar conclusion, identifying PALB as an independent prognostic factor for OS in an analysis of prognostic risk factors in 95 patients with triple-negative breast cancer (16). Subsequently, researchers developed a series of predictive models and indices based on PALB. Xu and his team collected data from 768 patients with advanced hepatocellular carcinoma (HCC) and developed a prognostic nomogram based on PALB. The C-index of their nomogram was 0.717 in the training cohort and 0.734 in the validation cohort, demonstrating high accuracy (17). Similarly, Zhang and his team constructed a prognostic nomogram based on PALB in a cohort of 881 patients with early-stage HCC, achieving similarly high C-index values (18).

Lymphocyte count is a key indicator for evaluating the immune status of the host. Lymphocytes play a critical role in antitumor immunity, and a decline in their levels may indicate immunosuppression or tumor-induced immune escape mechanisms (19). Additionally, LYM is highly responsive to the body’s inflammatory state. Inflammation plays a pivotal role in the initiation, progression, and metastasis of tumors, and persistent systemic inflammation can lead to lymphocyte depletion or dysfunction, thereby impairing the host’s antitumor response (20). Low lymphocyte levels are not only associated with inflammatory diseases but are also significantly linked to poor prognosis in various malignancies (21). LYM is a critical component of several prognostic indices, including PNI, NLR, and PLR, all of which have demonstrated substantial prognostic value in numerous previous studies (22–24). In 2022, Sun and his team collected clinical and pathological data from 146 gastric cancer patients who received ICIs (25). They not only found that PNI could effectively predict prognosis but also identified PALB as an independent prognostic factor in their study. The nomogram incorporating both PNI and PALB demonstrated excellent predictive accuracy. Another study investigated the adverse prognostic factors in HCC patients. They collected and analyzed data from 288 HCC patients who underwent liver resection and found that high LYM levels could reduce the risk of poor progression after liver resection in HCC patients (26). In 2024, a prospective study involving 1,449 patients with non-Hodgkin lymphoma (NHL) used machine learning to develop an efficient prognostic model. After minimizing confounding factors and eliminating the impact of multicollinearity, LYM was still found to be an independent prognostic factor for NHL patients, making it suitable for use in predictive modeling (27). On the other hand, researchers are also concerned about the application of LYM in COVID-19 patients. A study analyzing 109 COVID-19 patients found that LYM and IL-6 levels are closely related to the severity of the patients and are independent predictors of their mortality (28).

In this study, IgM demonstrated superior prognostic value as the key component of RIPI compared to other immunoglobulins. Its pentameric structure enables high-efficiency antigen binding and complement activation with C1q affinity 1,000 times stronger than IgG, directly destroying tumors through membrane attack complexes while initiating adaptive immunity (29). Unlike IgG, which is influenced by chronic inflammation, or IgA, which fluctuates with mucosal immunity, IgM’s short half-life (5 days) reflects real-time antitumor immune activity (30). Changes in IgM levels mirror immediate immune status, where increases correlate with effective immune coordination and decreases suggest immune exhaustion or microenvironment suppression (31). IgM uniquely reprograms immunosuppressive environments via FcμR-driven M1 macrophage activation and Treg/MDSC inhibition, mechanisms unaffected by tumor evasion strategies that disrupt IgG-mediated cytotoxicity (32). Combined with its stability against glycoform interference seen in IgG/IgA, these properties establish IgM as an essential biomarker for precise immune monitoring and prognostic stratification. As early as 1998, Tsavaris and his team investigated the significance of various circulating immunoglobulins in gastric cancer. After comparing immunoglobulin levels at different time points in 60 chemotherapy-naïve gastric cancer patients, they found that higher levels were associated with longer survival (33). Another gastric cancer study analyzed the prognostic value of immunoglobulins in patients receiving ICIs. After examining IgA, IgG, and IgM, the researchers arrived at a conclusion similar to that of the present study, namely that IgM had the highest prognostic value among circulating immunoglobulins (11). Similarly, Peppas and his team explored the roles of IgA, IgG, and IgM in bladder cancer and found that IgM had the greatest prognostic value (34).

The RIPI developed in this study, comprising PALB, LYM, and IgM, has demonstrated significant prognostic value. RIPI was not only significantly associated with both PFS and OS in survival analyses but was also identified as an independent prognostic factor for RCC patients. Its prognostic performance surpassed that of single indicators and multiple established indices. Additionally, after performing PSM analysis on RIPI, it still demonstrated high predictive value and was significantly associated with both PFS and OS, further highlighting its prognostic ability. Compared to single indicators or traditional prognostic indices, RIPI can assess patients’ immune function, inflammatory status, and nutritional levels in a multidimensional manner, making it more advantageous in prognosis prediction and personalized treatment decision-making. Single indicators are often influenced by various physiological factors, limiting their stability and accuracy. For example, although prealbumin reflects nutritional status, it is an acute-phase protein and is easily affected by inflammation. Even in well-nourished patients, chronic inflammation may lead to its reduction (35). Lymphocyte count primarily evaluates cellular immune function but can be significantly affected by factors such as infections, chemotherapy, and stress, causing short-term fluctuations (36). Immunoglobulin M, as a key component of humoral immunity, is influenced not only by B cell function but also by chronic diseases, immunosuppressive therapy, or coexisting infections (37). In contrast, RIPI integrates PALB, LYM, and IgM, three complementary indicators, reducing the impact of individual variability or external influences, thereby improving the stability and reliability of prognostic models. In renal cancer patients, this index not only distinguishes high-risk from low-risk patients with greater precision but also better predicts responses to immunotherapy, targeted therapy, and postoperative survival outcomes. Moreover, it provides a more scientific basis for dynamically monitoring treatment responses and disease progression, optimizing clinical management and personalized treatment strategies.

Although the RIPI developed in this study has shown high accuracy and clinical utility in predicting RCC prognosis, it has certain limitations. First, as a retrospective study, it may be subject to selection and information bias, requiring validation through large-scale prospective cohorts. Second, while RIPI integrates nutritional status, cellular immunity, and humoral immunity, its predictive performance may still be influenced by individual variability, tumor heterogeneity, and treatment factors. Differences in inflammatory status, immune response, and therapeutic regimens could affect its applicability across patient groups. Additionally, this study did not evaluate RIPI’s effectiveness under different treatment modalities, such as immunotherapy or targeted therapy. Further independent and multicenter studies are needed to refine RIPI and explore its potential in personalized treatment decision-making. Moreover, given that this study exclusively enrolled patients undergoing surgical resection, the distribution of TNM stages was inherently unbalanced, with a relative paucity of advanced-stage cases. This limited our ability to perform robust stratified analyses of RIPI across different disease stages. Future studies incorporating more diverse clinical cohorts are warranted to elucidate the stage-specific prognostic utility of RIPI.

## 5 Conclusion

The Renal Immune Prognostic Index (RIPI), consisting of PALB, LYM, and IgM, demonstrated strong prognostic value for renal cell carcinoma (RCC) patients. It effectively predicted clinical outcomes, including both progression-free survival and overall survival, and could serve as a valuable tool for guiding treatment strategies and improving personalized patient management in RCC.

## Data Availability

The raw data supporting the conclusions of this article will be made available by the authors, without undue reservation.
